# Custom-made fenestrated stent for mycotic aortic aneurysms: a report of two cases

**DOI:** 10.1186/s12872-021-02234-9

**Published:** 2021-09-10

**Authors:** Siting Li, Mengyin Chen, Yuehong Zheng, Zhili Liu, Rong Zeng

**Affiliations:** Department of Vascular Surgery, Peking Union Medical College Hospital, Chinese Academy of Medical Sciences and Peking Union Medical College, Beijing, 100730 People’s Republic of China

**Keywords:** Endovascular repair, Mycotic aneurysm, Paravisceral aortic aneurysm, Custom-made, Fenestrated stent-graft, Case report

## Abstract

**Background:**

Mycotic aortic aneurysm is a rare and potentially life-threatening lesion, and endovascular repair has become increasingly accepted for intervention. Fenestrated endografts are available options to treat aneurysms involving visceral arteries. Here, we first report two patients with mycotic aortic aneurysm involving paraviscereal aorta who were successfully treated with custom-made fenestrated endograft.

**Case presentation:**

Two patients were presented with mycotic aortic aneurysm. Due to their comorbidities and the involvement of the renal arteries, company-manufactured fenestrated stents were designed. Meanwhile, antibiotic therapy was administrated for 2 months before endovascular repair. Patients improved well without complications.

**Conclusions:**

Custom-made fenestrated endovascular stent is an effective and feasible alternative solution to mycotic paravisceral aorta aneurysm.

## Background

Mycotic aortic aneurysm (MAA) is a rare and potentially life-threatening lesion of the aortic wall characterized by a rapid growth rate and a high risk of rupture. It has a prevalence rate of 0.5–1.3% of aortic aneurysms in western countries, while could constitute up to 13.3% in Asia [1]. Golden standard management for MAA has been open surgical repair (OSR) with resection of the infected aortic segment and tissue debridement followed by revascularization. Endovascular aneurysm repair (EVAR) of MAAs, since first reported in 1998, has become increasingly accepted [2]. EVAR appears to be associated with improved short-term survival compared with OSR, with lower incidence of late disadvantages [3–5]. As a higher risk was associated with the surgical intervention of the aorta to maintain visceral perfusion, fenestrated EVAR (FEVAR) has been introduced. Custom-made fenestrated grafts are available options to treat aneurysms involving visceral arteries with satisfactory outcomes [6, 7]. In this report, we first presented two cases of MAA involving paravisceral aorta that has been successfully treated with company-manufactured, custom-made FEVAR.

## Case presentation

### Case 1

**Fig. 1:**
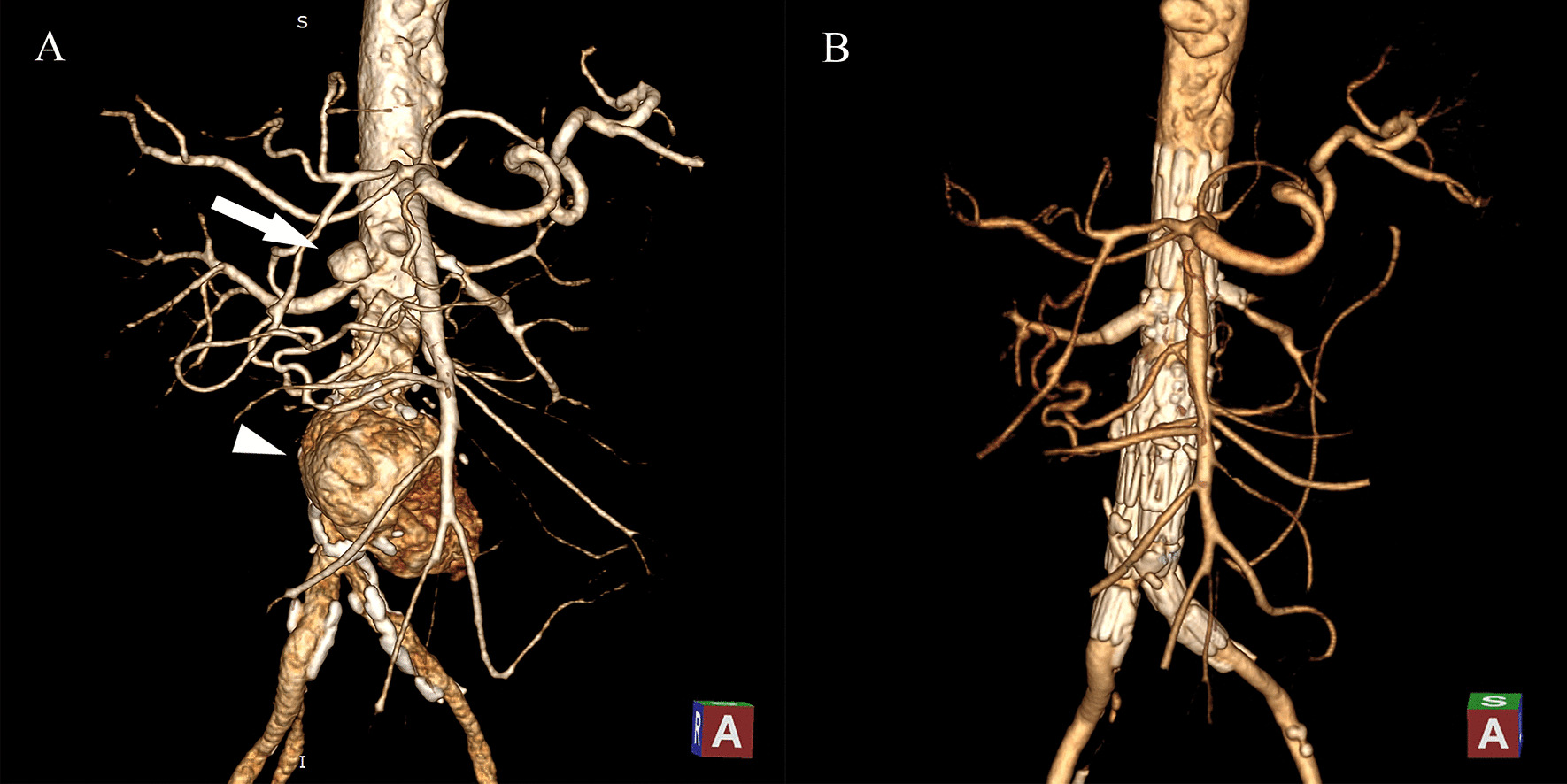
CT scans of the first patient. **A** Preoperative CT showed the renal aneurysm (white arrow) and the aortic aneurysm (white arrowhead). **B** 6-month follow-up CT scans

A 66-year-old man came to our hospital with 2 months history of undulant fever (up to 38.8 degrees Celsius), flank pain, and nausea. He had been diagnosed with hypertension, chronic obstructive pulmonary disease, and type 2 diabetes mellitus. Laboratory findings included significant elevation of inflammatory markers (C-response protein (CRP) 123.0 mg/L, procalcitonin (PCT) 0.87 ng/ml), and a positive Brucella agglutination test. Blood cultures for pathogens were negative. The computed tomography angiography (CTA) revealed aneurysms formation in the lower part of the abdominal aorta and the right renal artery (Fig. 1A). The thoracic aorta had transmural ulcers and the left internal iliac artery showed a severe occlusion.

Considering a diagnosis of mycotic aneurysm in the outpatient clinic, vancomycin (1 g, Q12h) and ceftriaxone (2 g, QD), and Ciprofloxacin (0.4 g, Q12h) were administered for ten days with remission of fever and pain. The patient was admitted to the vascular surgery ward. However, conservative treatment was chosen due to unstable temperature and diarrhea, and his antibiotics were adjusted to be minocycline (0.1 g, QD, p.o) and rifampicin (0.45 g, QD, p.o) on the advice of the antibiotic management team.

In view of the thoracic aortic ulcer and complicated condition of pseudoaneurysm in the abdominal aorta and right renal artery, a custom-made Zenith stent (Cook Medical, Bloomington, Ind) was constructed. The custom-made stent graft included small fenestrations for renal arteries and scallop for the superior mesenteric artery to reconstruct visceral arteries. Meanwhile, the patient remained on antibiotics and was admitted for surgery 2 months later.

**Fig. 2:**
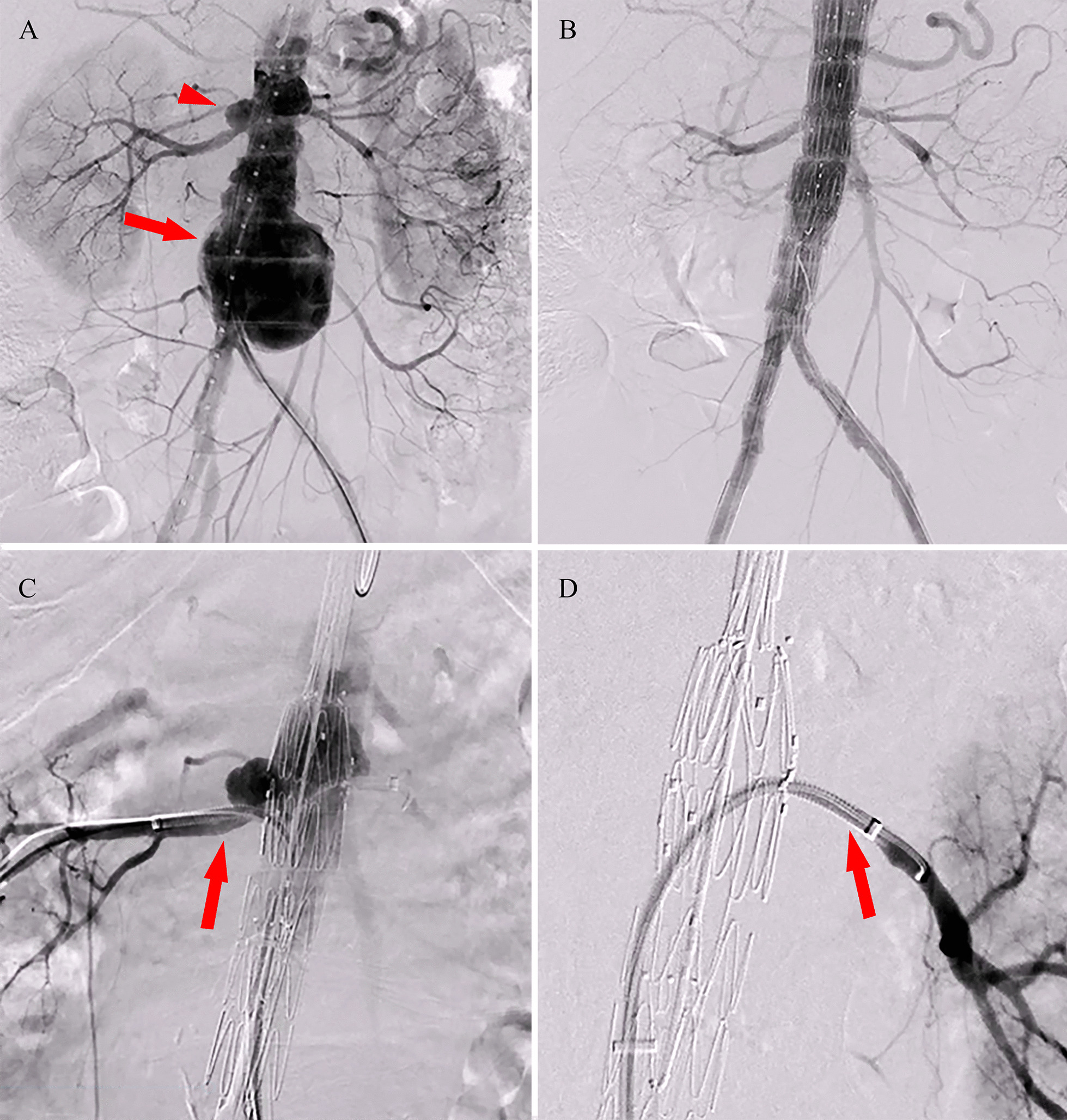
Angiography of the first patient **A** Preoperative angiography demonstrated the aneurysms of the abdominal aorta (red arrow) and right renal artery (red arrowhead); **B** Angiography after endovascular repair showed no endoleak or contrast extravasation; **C** Insertion of the right artery sheath (red arrow); **D** Insertion of the left artery sheath (red arrow)

Endovascular aneurysm repair (EVAR) was performed under general anesthesia. The proximal body of the custom-made stent was implanted according to the direction of the mark point. The marking points of the small fenestrations of both renal arteries were carefully observed to confirm the height of the stent by imaging. An 18 F vascular sheath was inserted through the left femoral artery, and 6 and 7 F vascular sheaths were placed in the sheath to establish renal artery access (Fig. 2C, D). A balloon-expanded stent (Boston Scientific Express LD) was placed in the left renal artery, while a covered stent (GORE VIABAHN) was introduced into the right. The contralateral iliac branch was implanted after the release of the distal body. The overlap area and seal area underwent balloon expansion after deployment. Postoperative angiography showed that the superior mesenteric artery, both renal arteries, and bilateral iliac branches were unobstructed with the aneurysm well isolated (Fig. 2B). The patient recovered well without fever or abdominal pain. Antibiotic therapy was continued for 3 months after discharge. At the 6-month follow-up assessment, CTA showed that the aneurysm disappeared completely and the abdominal aorta, iliac and renal artery stents were adequately filled with contrast medium (Fig. 1B).

### Case 2

**Fig. 3:**
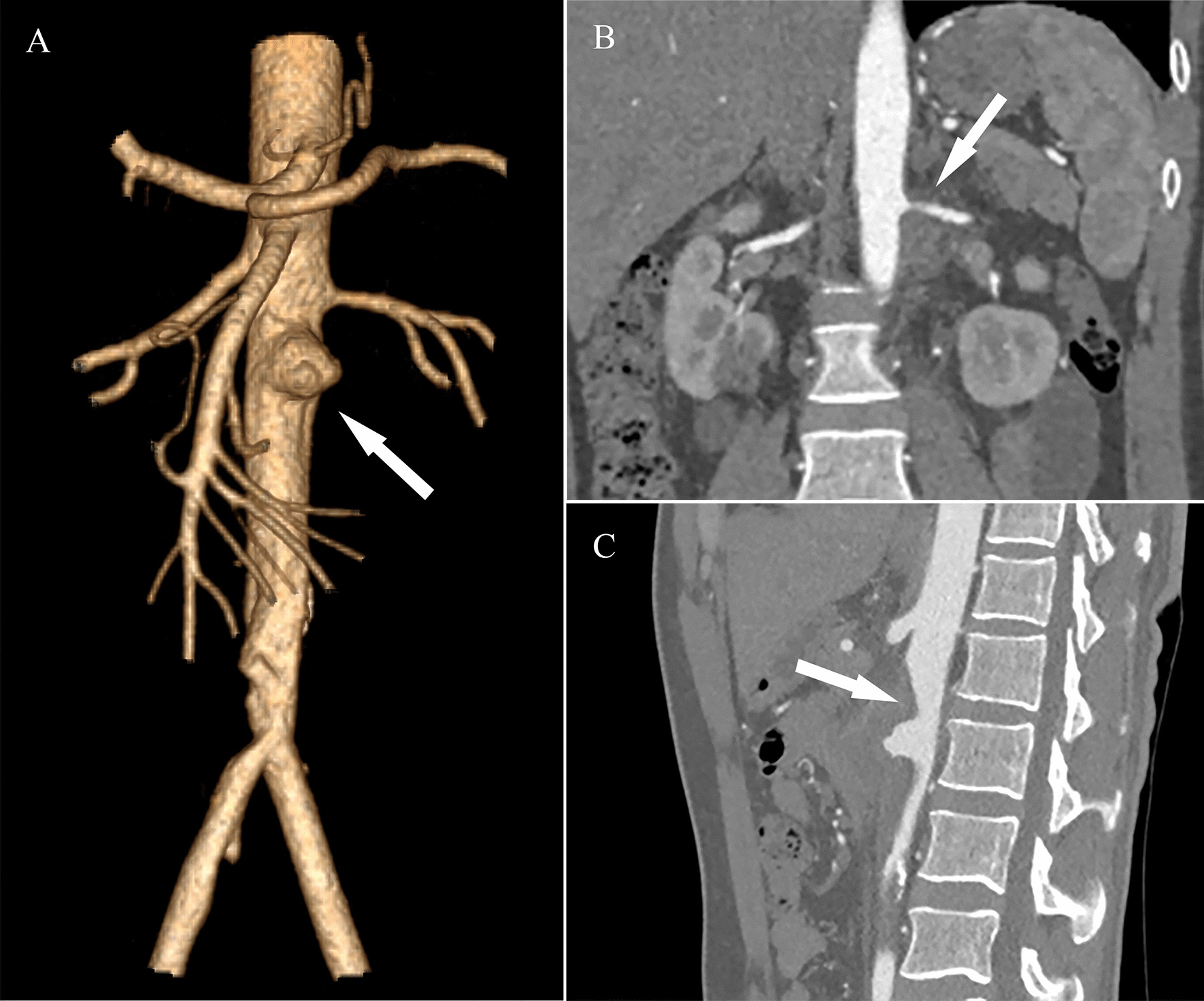
CT scans of the second patient **A** 3D reconstructions of CT scans shows aneurysm (white arrow); **B** Compression of the left renal artery and the lumen (white arrow); **C** Abdominal aorta is compressed and deformed by the aneurysm (white arrow)

A 36-year-old man who was suffering from recurrent fever and bilateral flank pain for 1 week was admitted to our hospital. He had a history of deep vein thrombosis. Laboratory values showed a significant increase in CRP (91.57 mg/L) and erythrocyte sedimentation rate (ESR; 60 mm/h). The 2-deoxy-2-[F18]-fluoro-D-glucose-positron-emission tomography/computed tomography (FDG-PET/CT) demonstrated an abdominal aortic aneurysm with a highly metabolic wall (SUVmax = 9.3). Blood cultures for pathogens were negative. CTA illustrated an aneurysm below the superior mesenteric artery, with the largest section of about 4.4 cm × 4.9 cm. Slightly high-density shadow with edge enhancement surrounded the aneurysm. The left renal artery is locally compressed and the lumen is slightly narrowed. Mural thrombus formed in the right common iliac artery and the lumen of the right internal iliac artery was severely narrowed (Fig. 3). Echocardiogram showed severe pulmonary hypertension (pulmonary artery systolic pressure (PASP) = 72 mmHg). A diagnosis of infected aneurysm was made. The patient was admitted to the vascular surgery ward, and treatment with antibiotics including ceftazidime and vancomycin was administered for 4 weeks. During antibiotic therapy, the inflammatory markers decreased gradually to CRP of 4.14 mg/L and ESR of 8 mm/h.

Due to the patient’s comorbidities and the compression of the renal artery, a custom-made Zenith stent was planned. The custom-made stent graft included small fenestrations for renal arteries and large fenestration for the superior mesenteric artery to reconstruct visceral arteries. Meanwhile, the patient remained on antibiotics ceftazidime (2 g q12h) and was admitted one month later.

**Fig. 4:**
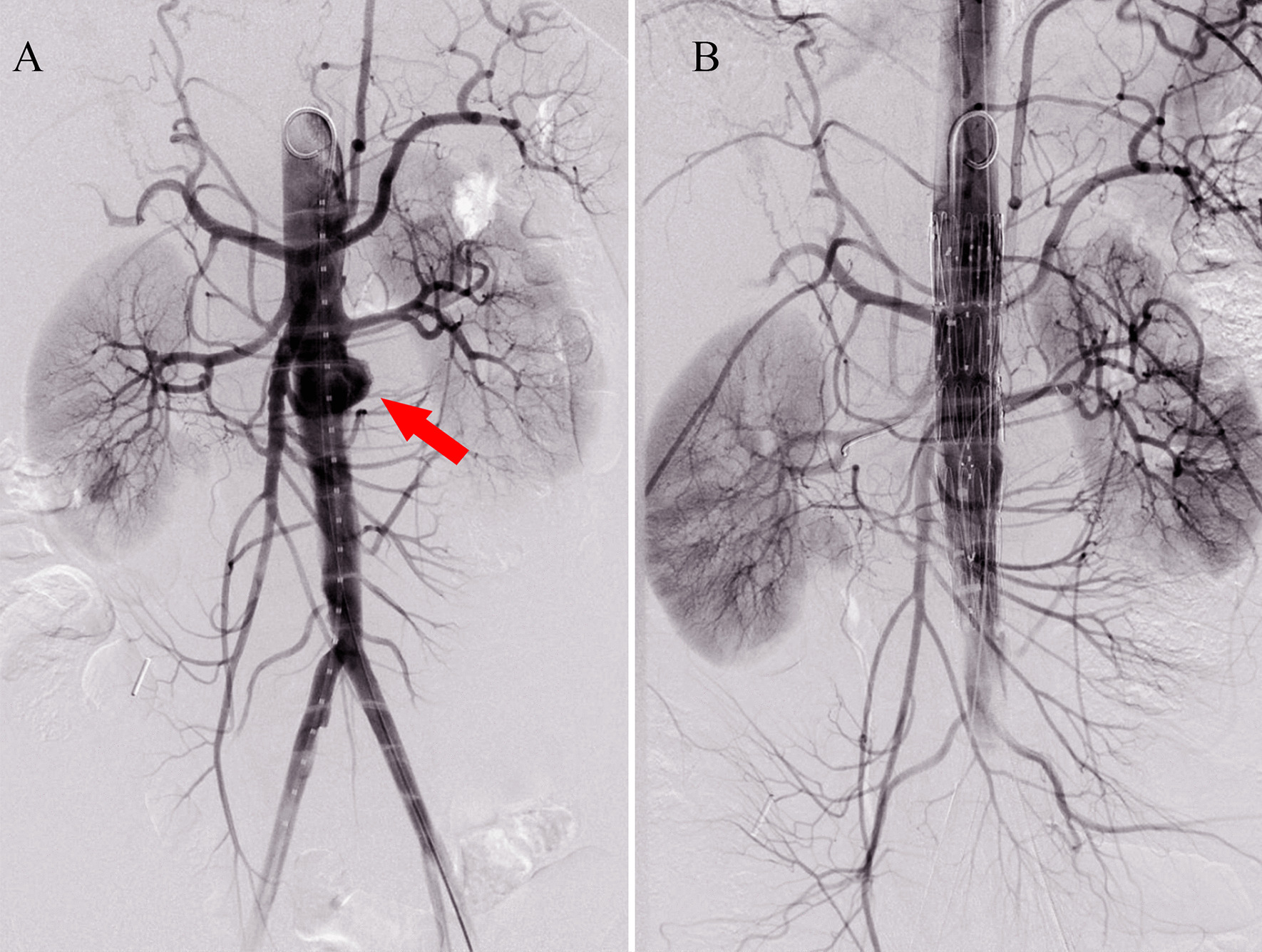
Angiography of the second patient **A** Preoperative angiography showed the aneurysms of the abdominal aorta (red arrow), **B** Final angiography showed no endoleak or contrast extravasation

EVAR was offered to the patient under general anesthesia. The final angiography showed that the renal arteries, superior mesenteric artery, and common iliac arteries were unobstructed, and no endoleak or contrast extravasation was observed (Fig. 4). After the operation, the patient continued antibiotic therapy for 3 months and improved well without complications.

## Discussion

Although EVAR is less invasive and suitable for high-risk patients, it had been questioned whether the remained infected tissue may cause stent graft infection and recurrent aneurysm. However, growing evidence in recent years has proved the efficacy of EVAR for MAAs. Sorelius et al. analyzed 10-year data on patients with MAAs in Sweden, which indicated that EVAR could improve short-term survival without increasing the risk of infection-related complications or reoperations [5]. A systematic review performed later by the team demonstrated that EVAR was the dominant surgical technique since 2011 and had better short-term survival than open surgery [8].

Nowadays, FEVAR is becoming a widely available option in the management of aneurysms involving visceral arteries with satisfactory short and mid-term outcomes. Gallitto et al. used custom-made and off-the-shelf endografts in 60 and 28 patients with thoracic abdominal aortic aneurysm (TAAA) respectively. Survival at 1 year was 86 % and at 2 years was 68 %. The patency of target visceral vessels at 2 years was 92% [9]. A recent publication from Niet et al. reported favorable results in 335 patients with complex AAA which included short neck, juxtarenal and thoracoabdominal aortic aneurysm. The target vessel patency at one and three years were 96.4 and 92.7%. The median follow-up was 1.2 years and 53 patients (15.8%) died during follow-up [10].

Physician-modified endografts (PMEG) and company-manufactured devices (CMD) are two available sources for custom-made FEVAR. Regarding MAAs, several cases have been reported utilizing PMEG. To treat a saccular mycotic aneurysm in the suprarenal aorta, Sule et al. created fenestrations for the coeliac artery and superior mesenteric artery and lined each fenestration with soft nitinol wire. The patient remained well without signs of infection during the 1-year follow-up interval [11]. Durgin et al. used physician-modified endograft to treat a large, contained rupture of a juxtarenal AAA. 6-month follow-up CT showed that the aneurysm had completely regressed [12]. In a recent case, Satoshi et al. conducted stent fenestrations for the celiac artery, superior mesenteric artery, left and right renal arteries for a patient suffering from mycotic multiple aortic aneurysms. Follow-up CT at 2 years and 4 months indicated shrinkage and disappearance of aneurysms [13].

Previous studies have suggested a reduction of mortality and re-intervention rate for CMD compared to PMEG [14, 15]. PMEG may offer acceptable results in patients requiring urgent interventions, yet required experience and proficiency of the physicians [16]. CMD has the advantages of greater quality control and a broader range of options for fenestrations, while time delay for device manufacturing is its major constraint. In the case of mycotic aortic aneurysms, retrospective analysis of 40 patients has revealed that 75% of the patients with persistent infection died within 12 months [17]. Thus, adequate antimicrobial treatment, both pre-and post-operatively, is a key prerequisite for successful endovascular repair. Patients who are relatively sterile at the time of stent graft deployment had been shown to have fewer long-term infective complications [18]. The period of antibiotic therapy can be utilized to construct commercial custom-made stents, especially for patients with complicated paravisceral aorta anatomies and relative stable hemodynamics. Long-term antibiotic therapy for at least 6 to 12 months and possibly for life is also recommended after the operation to prevent recurrent infections [5].

## Conclusions

Two patients with mycotic aortic aneurysm involving paravisceral aorta were treated with company-manufactured, custom-made endovascular stents and recovered well without complications. Custom-made fenestrated stent is an effective and feasible alternative solution to mycotic paravisceral aorta aneurysm. Long-term surveillance of stents efficacy as well as freedom of infection is still required for the patients, and larger studies would be needed in the future to confirm these data.

## Data Availability

All relevant data supporting the conclusions of this article are included within the article.

## Abbreviations

MAA: Mycotic aortic aneurysm
OSR: Open surgical repair
EVAR: Endovascular aneurysm repair
FEVAR: Fenestrated endovascular aneurysm repair
CRP: C-response protein
PCT: Procalcitonin
CTA: Computed tomography angiography
ESR: Erythrocyte sedimentation rate
FDG-PET/CT: 2-deoxy-2-[F18]-fluoro-D-glucose-positron-emission tomography/computed tomography
PASP: Pulmonary artery systolic pressure
TAAA: Thoracic abdominal aortic aneurysm
PMEG: Physician-modified endografts
CMD: Company-manufactured devices
